# A Multinational Analysis of Mutations and Heterogeneity in PZase, *RpsA*, and *PanD* Associated with Pyrazinamide Resistance in M/XDR *Mycobacterium tuberculosis*

**DOI:** 10.1038/s41598-017-03452-y

**Published:** 2017-06-19

**Authors:** S. M. Ramirez-Busby, T. C. Rodwell, L. Fink, D. Catanzaro, R. L. Jackson, M. Pettigrove, A. Catanzaro, F. Valafar

**Affiliations:** 1 Biological and Medical Informatics Research Center, San Diego State University, San Diego, California, USA; 2 Department of Medicine, University of California, San Diego, California, USA; 3 Department of Biological Sciences, University of Arkansas, Fayetteville, Arkansas USA

## Abstract

Pyrazinamide (PZA) is an important first-line drug in all existing and new tuberculosis (TB) treatment regimens. PZA-resistance in *M. tuberculosis* is increasing, especially among M/XDR cases. Noted issues with PZA Drug Susceptibility Testing (DST) have driven the search for alternative tests. This study provides a comprehensive assessment of PZA molecular diagnostics in M/XDR TB cases. A set of 296, mostly XDR, clinical *M. tuberculosis* isolates from four countries were subjected to DST for eight drugs, confirmatory Wayne’s assay, and whole-genome sequencing. Three genes implicated in PZA resistance, *pncA*, *rpsA*, and *panD* were investigated. Assuming all non-synonymous mutations cause resistance, we report 90% sensitivity and 65% specificity for a *pncA*-based molecular test. The addition of *rpsA* and *panD* potentially provides 2% increase in sensitivity. Molecular heterogeneity in *pncA* was associated with resistance and should be evaluated as a diagnostic tool. Mutations near the N-terminus and C-terminus of PZase were associated with East-Asian and Euro-American lineages, respectively. Finally, Euro-American isolates are most likely to have a wild-type PZase and escape molecular detection. Overall, the 8–10% resistance without markers may point to alternative mechanisms of resistance. Confirmatory mutagenesis may improve the disconcertingly low specificity but reduce sensitivity since not all mutations may cause resistance.

## Introduction

Pyrazinamide (PZA) is an important first-line drug recommended by the World Health Organization (WHO) for treatment of tuberculosis (TB)^1^. Although most patients diagnosed with TB are successfully treated with first-line drugs, rifampicin (RIF), isoniazid (INH), ethambutol, and PZA, the incidence of multidrug resistant TB (MDR-TB) (resistant to first-line drugs) is increasing every year^1^. Along with this rate, the incidence of PZA resistance (PZA^R^) is also increasing since over 60% of MDR-TB cases globally are also PZA^R^ (rates vary regionally)^2^. In this manuscript we denote isolates that are resistant or susceptible to PZA by growth-based drug susceptibility testing (DST) as PZA^R^ or PZA^S^, respectively.

PZA is unique among anti-TB drugs in that, while it demonstrates powerful *in vivo* sterilizing activity, it exhibits no activity against actively growing *Mycobacterium tuberculosis* bacilli under normal culture conditions at neutral pH^3^. The drug appears to preferentially act against non-replicating persisters with low metabolic activity at acid pH *in vitro* or *in vivo* ^4^.

Importantly, most new drug regimens proposed for treatment of drug-resistant TB (DR-TB) show improved outcomes when combined with PZA^5–9^. Although growth-based PZA susceptibility testing is recommended by the WHO^10^, the method yields inconsistent results in some cases and suffers from a relatively high false resistance rate^11^.

While DST is the recommended method for determining resistance to all first- and second-line drugs^1^, because of PZA-specific challenges, WHO is currently considering *pncA*-based molecular diagnostics as the recommended approach. In this study we examine the performance of such a platform in detection of PZA resistance among M/XDR TB cases from four high-TB burden countries. We also examine how the inclusion of the other two frequently discussed genes, *panD* and *rpsA*, would improve the proposed platform.

The protein commonly associated with PZA resistance is pyrazinamidase/nicotinamidase (PZase) encoded by the gene *pncA* ^12^. Mechanistically, PZA is a pro-drug that requires activation by PZase. PZase converts PZA into pyrazinoic acid (POA), where it is actively driven out of the cell, extracellularly protonated, and passively diffused back in, eventually acidifying and killing the bacterium^4, 13^. Hundreds of mutations distributed across *pncA* and its promoter have been associated with resistance, however some are also harbored by susceptible strains^14, 15^. This is unique since resistance to other drugs can usually be explained by a handful of mutations^16–18^. A recent systematic review estimated mutations in *pncA* and its promoter provide a global sensitivity and specificity of 83% and 90%, respectively^14^.

While changes in PZase have been associated with PZA resistance, several studies have observed PZA^R^ isolates with a wild-type (WT) *pncA* ^19–23^, highlighting the need for discovery of new markers that could be used in molecular diagnostics. Recently, mutations in 30 S ribosomal protein S1 (*rpsA*) and aspartate 1-decarboxylase precursor (*panD*) have been reported to be associated with PZA resistance in *M. tuberculosis* ^24, 25^.

The gene *rpsA* encodes a protein involved in trans-translation, a mechanism that rescues stalled ribosomes and tags truncated proteins for degradation^24^. Zhang *et al*. demonstrated that overexpression of *rpsA* in *M. tuberculosis* confers PZA resistance *in vitro* ^24^.

The gene *panD* converts L-aspartate into β-alanine, a precursor in the anabolism of coenzyme A (CoA)^25^. Laboratory generated strains of PZA^R^ *M. tuberculosis* were observed with mutations in *panD*, yet WT-*pncA* ^25^. However, subsequent studies have rebutted *rpsA* and *panD* as potential targets of PZA resistance^26, 27^.

In this study, we considered 296 mostly M/XDR-TB patients from four high-burden TB countries: India, Republic of Moldova, the Philippines, and South Africa. Unlike previous systematic reports, we have ensured standardized phenotyping for eight first and second line drugs, including PZA, using BACTEC MGIT 960 DST^28^. Additionally, we have performed PZase enzymatic activity testing using Wayne’s assay to confirm DST results. Genotyping was also performed via amplification-free long-read whole-genome sequencing for each isolate. Mutations in *pncA*, *rpsA*, and *panD* and their promoters were investigated for association to phenotypic PZA resistance.

## Results

### Phenotypic testing

Out of 296 mostly XDR-TB isolates (Table 1), 224 were PZA^R^ and 72 were PZA^S^ by DST. Two isolates were PZA mono-resistant. Table 1 also displays the prevalence of PZA resistance in all phenotypic categories.

**Table 1:** Drug susceptibility patterns per lineage among the GCDD *M. tuberculosis* clinical isolates. Numbers in parentheses indicate total isolate counts for each category.

PZA Phenotype	Lineage	Pan-Susceptible	Mono	MDR	Pre-XDR	XDR	Other
**All (296)**	All (296)	19	8	16	36	207	10
**Resistant (224)**	All Lineages (224)	0	3	11	26	179	5
	East Asian (Beijing) (115)	0	0	1	10	103	1
	East Asian (1)	0	0	0	1	0	0
	Indo-Oceanic (18)	0	1	6	2	6	3
	Euro-American (81)	0	1	4	13	63	0
	East-African Indian (CAS) (9)	0	1	0	0	7	1
**Susceptible (72)**	All Lineages (72)	19	5	5	10	28	5
	East Asian (Beijing) (21)	2	1	1	4	12	1
	East Asian (0)	0	0	0	0	0	0
	Indo-Oceanic (16)	6	3	2	4	0	1
	Euro-American (31)	9	1	1	2	16	2
	East-African Indian (CAS) (4)	2	0	1	0	0	1

**PZA**: pyrazinamide; **MDR-TB:** resistant to isoniazid and rifampicin, only; **XDR-TB:** MDR-TB that is also resistant to at least one fluoroquinolone and one injectable; **pre-XDR-TB:** MDR-TB and resistant to either a fluoroquinolone(s) or an injectable(s); **Pan-Susceptible:** susceptible to all seven (INH, RIF, CAP, AMK, KAN, OFX, MOX) drugs tested; **Mono:** isolates resistant to only one of the seven study drugs; **Other:** isolates with unusual phenotypic patterns such as susceptibility to INH but resistance to second line drugs.

In all, 47 (16%) strains (22 PZA^R^ and 25 PZA^S^) had discordant phenotypic-genotypic results (i.e. PZA^S^ with a mutant *pncA* or promoter, or PZA^R^ with a WT *pncA* or promoter—Supplementary Table ST1) and underwent further enzymatic characterization.

### Enzymatic activity

Overall, 103 isolates (79 PZA^R^ and 24 PZA^S^), including 44 (22 PZA^R^ and 22 PZA^S^) of the 47 phenotypic-genotypic discordant isolates, were tested for enzymatic activity (Table 2). Of the 103 tested isolates, five (5%) (one PZA^R^ and four PZA^S^) had discordant DST and Wayne’s assay results. The DST results of 41 of the 44 phenotypic-genotypic discordant isolates were confirmed by Wayne’s assay. Only three PZA^S^ isolates had a discordant DST and enzymatic activity results (Table 2).

**Table 2:** Results of the Wayne**’**s pyrazinamidase (PZase) activity assay for 103 (including 44 *pncA* genotypic-phenotypic discordant) clinical *M. tuberculosis* isolates. Drug susceptibility was determined by BACTEC MGIT 960. Numbers in parentheses indicate the number of isolates with discordant PZA phenotype and *pncA* genotypic (i.e. resistant isolates with WT *pncA* and promoter or susceptible isolates with mutant *pncA* or promoter).

PZA DST Result	PZase Positive	PZase Negative
Resistant	1 (0)	78 (22)
Susceptible	20 (19)	4 (3)

### Mutations in *pncA* of PZA^R^ Isolates

Of the 224 PZA^R^ isolates, 202 harbored a non-synonymous mutation in *pncA* and/or its promoter. Of these, 195 isolates (87%) harbored a mutation only in the gene, four (2%) only in its promoter, and 3 (1%) in both. The two PZA mono-resistant isolates belonged to the first group. Synonymous mutations in the coding region were ignored in our analyses. Those isolates that only harbored a synonymous mutation in a gene were labeled as having a WT protein in this study. Supplementary Table ST2 provides a comprehensive list of all mutations harbored by all isolates. Importantly, 22 PZA^R^ isolates (10%) had a WT *pncA* gene and promoter. All 22 tested negative for PZase activity (Table 2 and Supplementary Table ST2). These 22 isolates were not part of the same clonal expansion (based on the lineage typing) (Supplementary Table ST2).

In 224 PZA^R^ isolates, we observed 136 unique protein-altering polymorphisms in *pncA* and 6 unique mutations in its promoter. Of these, 40 polymorphisms in the gene and three (all indels) in the promoter had not been previously reported and are referred to here as novel mutations (Supplementary Table ST3)^14^.

**Figure 1:**
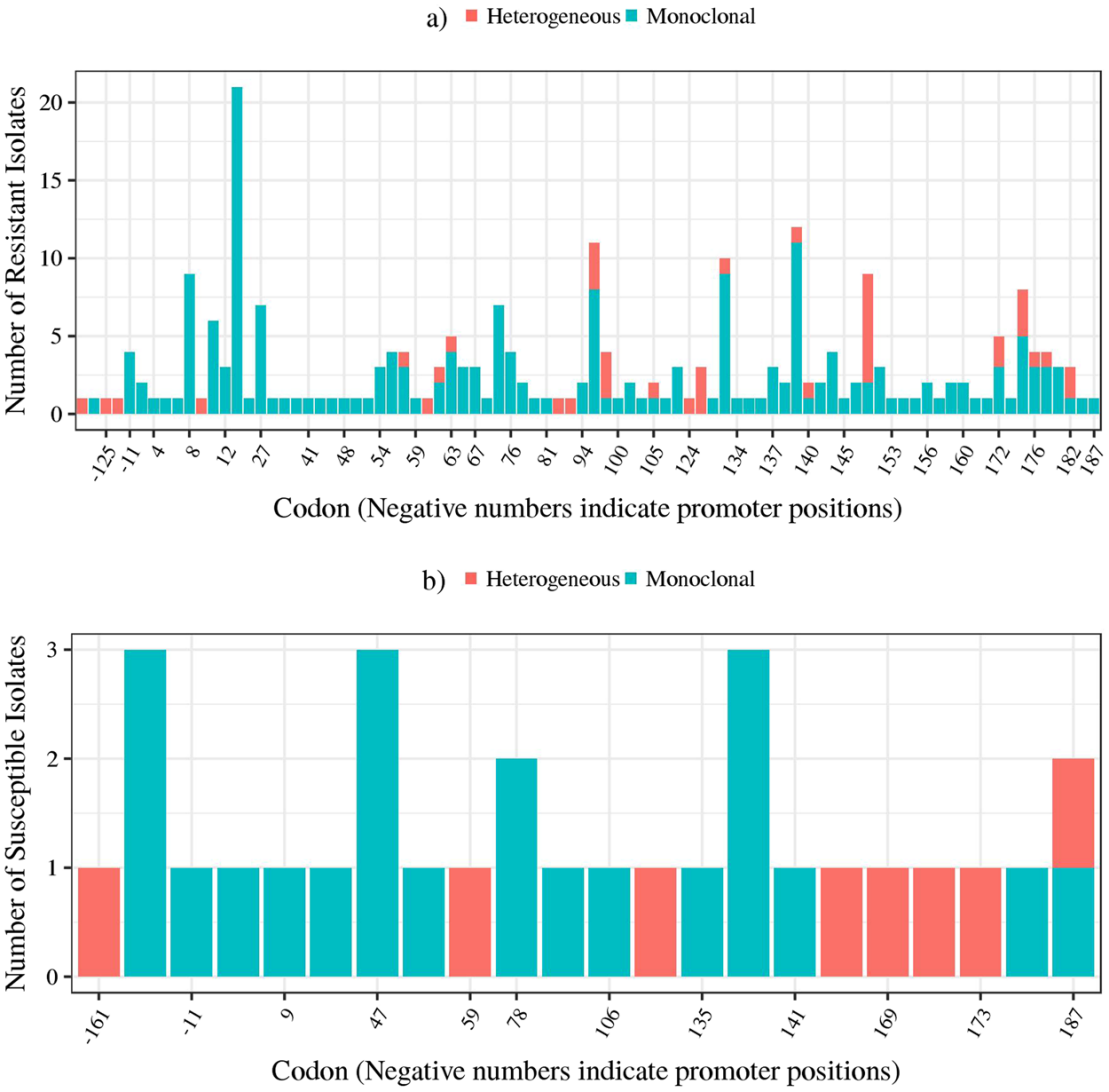
Distribution of *pncA* mutations in (**a**) PZA^R^ and (**b**) PZA^S^ clinical *M. tuberculosis* isolates. Frequencies labeled as “Heterogeneous” represent calls that had sufficient support for both a variant and the reference, indicative of mixed populations. “Monoclonal” frequencies represent calls that were clearly supportive of a variant. The frequencies presented here are the totals over all the different mutations observed at each codon.

The distribution of mutations across the gene is shown in Fig. 1. The most variable locus was codon 14 (Fig. 1a). Two variants were observed in the codon, Cys14Arg and Cys14STOP. Cys14Arg was the most frequently observed mutation (20 isolates from South Africa). Eighteen had identical MIRU-VNTR and spoligotyping patterns suggesting a clonal expansion (East-Asian, Beijing sublineage). Cys14STOP was observed in one isolate from Moldova and belonged to the Euro-American lineage.

### Mutations in *pncA* of PZA^S^ isolates

Of the 72 susceptible isolates, 25 harbored a mutation in the coding region of *pncA* and/or in its promoter (Table 3 and Fig. 1b). Twenty-six (22 coding and 4 promoter) unique mutations in *pncA* of PZA^S^ isolates were observed. Of the coding region mutations, 13 were novel, and of the promoter mutations, 2 were novel (Supplementary Table ST4). The most frequent non-synonymous polymorphism was Thr47Ala, occurring in three PZA^S^ isolates, all of which tested positive for PZase activity (Fig. 1b).

**Table 3:** Frequency of mutations in *pncA* and its promoter, *panD* and its promoter, and *rpsA* and its promoter in *M. tuberculosis* clinical isolates.

Category	pncA	panD	rpsA
PZA^R^ with mutant enzyme*	195	7	19
PZA^R^ with mutant promoter (but WT enzyme^†^)	4	1	1
PZA^R^ with mutant promoter and enzyme	3	0	0
PZA^R^ with WT enzyme^†^ and promoter	22	216	204
PZA^S^ with mutant enzyme*	19	1	7
PZA^S^ with mutant promoter (but WT enzyme^†^)	6	2	1
PZA^S^ with mutant promoter and enzyme	0	0	0
PZA^S^ with WT enzyme^†^ and promoter	47	69	64

WT = wild-type.

*Includes heterogeneous variations (i.e. mixed populations with mutant and WT gene/promoter).

^†^Also includes isolates that harbor synonymous mutations (only) in the gene.

### Lineage-based Analysis of *pncA* mutations

Supplementary Figure SF1 depicts lineage-based stratification of mutations observed in *pncA*, both in resistant and susceptible isolates. The gene and its promoter were divided into seven “zones” and prevalence of mutations in isolates from each lineage was assessed. The results of this analysis are shown in Supplementary Table ST5. Most notable was the relatively high percentage (15%) of Euro-American PZA^R^ isolates with WT *pncA* and promoter. Furthermore,”hot spots” were observed in specific lineages: codons 1–30 for East-Asian, 121–150 for Indo-Oceanic, and codons 151 and higher for Euro-American. Similarly, there appears to be “cold spots” (31–60 for East-Asian and codons 91–120 for Euro-American), where very few isolates from these lineages harbored a mutation. Finally, the range between codons 91 and 120 seems to be a cold spot for all lineages, except for East-Asian. These patterns need to be confirmed in larger cohorts.

### Mutations in *rpsA*

Twenty PZA^R^ and eight PZA^S^ isolates harbored a mutation in RpsA (18 PZA^R^, seven PZA^S^) or *rpsA*’s promoter (2 PZA^R^, 1 PZA^S^) region (Supplementary Table ST2). Of these 14 PZA^R^ and 4 PZA^S^ isolates only had heterogeneous mutations in the *rpsA* and its promoter. Of the 22 resistant isolates without a mutation in *pncA* or its promoter, three had a heterogeneous, non-synonymous mutation in *rpsA* (deletion of C in nucleotide 660, deletion of a C in nucleotide 1065 [novel], and deletion of a C in nucleotide 1142 [novel]). The three showed no PZase activity on Wayne’s assay and had no mutations in *panD*. The most frequent *rpsA* variant was the previously reported synonymous change, Arg212Arg (99 PZA^R^ and 20 PZA^S^ isolates)^26^. This mutation has been identified as a Lineage 2 (East-Asian) marker. In our set, this mutation was harbored by three Euro-American isolates as well (Supplementary Table ST6)^29^.

### Mutations in *panD*

Previous studies have reported *panD* to be a potential target for PZA^25, 30^. In this study, no monoclonal mutations were found in *panD*’s promoter or coding region. Of the PZA^R^ isolates without a mutation in *pncA*, one had a heterogeneous mutation in *panD* (-G291) (Supplementary Table ST2). This isolate showed no PZase activity.

### Resistant Cases with WT promoter and coding regions in the three genes

Eighteen PZA^R^ isolates had no mutations in the promoter or the enzyme of the three genes considered in this study. All tested negative for enzymatic activity. Nine of the 18 belonged to Euro-American lineage, while eight were East-Asian (Beijing), and one was Indo-Oceanic.

### Heterogeneous Populations in *pncA*

The mutation counts reported here also include heterogeneous variants. Mixed populations were detected in *pncA* (coding and promoter region) of 40 isolates (34 PZA^R^, 6 PZA^S^) (Supplementary Tables ST7 and ST2). In 13 of the 34 PZA^R^ isolates, the heterogeneous variant was the only polymorphism in the three genes. Nine heterogeneous variants were observed in the 13 isolates (Supplementary Table ST8). The most frequent of these variants was the novel insertion of C in nucleotide 453 which was observed in five resistant isolates. Three belonged to East-Asian, three belonged to Indo-Oceanic, two belonged to CAS, and one was Euro-American, ruling out the possibility of clonal expansion. In *rpsA* (coding and promoter region), 21 isolates (16 PZA^R^, 5 PZA^S^) had heterogeneous calls, and in *panD*, 11 isolates (8 PZA^R^, 3 PZA^S^) exhibited this behavior. No isolate had heterogeneity in more than one of the studied genes. The frequency of a heterogeneous observation in *pncA* of PZA^R^ isolates was notably higher than that of PZA^S^ isolates (15% resistant versus 8% susceptible). These frequencies were (7% versus 7%) for *rpsA* and (4% versus 4%) for *panD* (Supplementary Table ST7).

## Discussion

This multinational study is based on strains collected from pulmonary TB patients in four high burden countries. Our primary objective was to determine the accuracy of a molecular test to diagnose PZA resistance in our set of MDR- and XDR-TB patients from high TB burden regions. Three genes, *pncA*, *rpsA*, and *panD*, were considered in this study. Although each gene has been well-studied independently, a concurrent assessment of all three in MDR- and XDR-TB patients from multiple high-burden countries has been limited.

### Correlation with resistance to other drugs

Among our isolates, broader resistance to other drugs directly translated to higher prevalence of PZA resistance. None of our isolates that were susceptible to the other seven drugs were PZA^R^, while 38% of those mono resistant to one of the other drugs, 69% of MDR isolates, and 86% of XDR isolates were PZA^R^ (Supplementary Figure SF2). While it is known that PZA resistance is associated with MDR status^2^, such a direct relationship to the breadth of resistance to second line drugs, beyond MDR, is less established and should be investigated further in larger cohorts.

### Phenotypic accuracy

Both false resistance and susceptibility have been noted for MGIT PZA DST in literature. The prevalence of false resistance, however, has been reported to be unusually high^11^. Over-inoculation is a common cause of false resistance and is a frequently the suspected reason for phenotypic-genotypic discordance^31, 32^. Using orthogonal confirmation (see the Phenotyping section in Methods), we confirmed lack of enzymatic activity in all 22 phenotypic-genotypic discordant PZA^R^ isolates (with WT *pncA* and promoter) (Table 2). We were also able to establish the presence of enzymatic activity in 19 of the 22 phenotypic-genotypic discordant PZA^S^ isolates (with a mutant *pncA*) (Table 2). Overall, we observed 95% concordance between DST and enzymatic activity (Cohen’s Kappa = 0.86). (Table 2) Majority of discordant cases (4 out of 5) were susceptible isolates with a mutant *pncA*. We hypothesize that this relates to mutations that do not cause a disruption in enzymatic activity or do so minimally causing low level resistance undetectable by the current cutoff. (Please see the sections on Sensitivity/Specificity and Causation).

### Novel pncA mutations

Overall 43 of the 142 (30%) unique variants observed in *pncA* of our PZA^R^ isolates were previously not reported. (Supplementary Table ST3) Similarly, 15 of the 26 (58%) variants observed in *pncA* of our PZA^S^ isolates were novel. The high percentage of novel *pncA* variants is not unusual. The variable nature of the gene is well documented and hundreds of unique mutations have already been reported^2, 14, 23, 33^. The notably higher rate of novel variants observed in PZA^S^ isolates is new. This may reflect a bias toward sequencing resistant isolates in the past—a practice that needs to change since an accurate catalog of mutations not associated with resistance is essential for molecular diagnostics. Among others, Whitfield *et al*. reported 10 *pncA* mutations that do not confer PZA resistance at the cut off of 100 mg/L^34^. Here, we add 15 more polymorphisms to the list (Supplemental Table ST4).

### Sensitivity/specificity

A wide range of sensitivities have been regionally reported for *pncA* mutations, ranging from 45.7% in Rio de Janeiro^35^ to 93% in China^36^. We report 90.2% (202/224) concordance between *pncA* genotype and phenotype among our resistant isolates. This is in line with some previous reports^11, 26, 37^, and higher than the global sensitivity of 83%^14^. A wide range of specificities has also been reported in the literature. For example, Osman *et al*. reported a specificity of 63% for *pncA* mutations^38^, while, Juréen *et al*. reported a specificity of 97.3% in China^36^. We report a specificity of *pncA*-based diagnostics in our study at 65% (25/72 PZA^S^ isolates had a mutant *pncA* or promoter) which is notably lower than the global specificity of 90%^14^, but within the reported regional ranges^14, 23, 39^. It is known that not all changes affect the function of the enzyme significantly. The lower specificity in our study is due to the large number of novel *pncA* polymorphisms observed in our PZA^S^ isolates. Since the phenotype has been confirmed by the enzymatic activity, as mentioned, we believe that these mutations either do not change the function of the enzyme or do so minimally causing low levels of resistance below MGIT cutoff.

An important note about the sensitivity and specificity reported here is that these percentages depend on the criteria used for their calculation. Several such criteria have been proposed in the literature. The most common, and one that we have used, employs all *pncA* promoter or PZase variants as a marker for resistance. This provides the highest sensitivity but low specificity. Alternatively, all mutations observed in susceptible isolates could be excluded, providing 100% specificity but dramatically reducing sensitivity from 90% to 65% (146/224) in our study. Miotto *et al*.^23^ propose a few different criteria, all of which would produce percentages between the two extremes calculated here. For instance, excluding mutations over 20 base pairs transcriptionally upstream of the gene would improve specificity from 65% to 70% but reduce sensitivity below 90%. The choice of this criterion will have important implications for molecular diagnostic platforms.

### Lineage trends

Several trends can be noted in Supplementary Table ST5. Perhaps the one bearing most significance is that the Euro-American lineage is the most likely to escape molecular diagnostics, as it has the highest percentage (15%) of resistant isolates without a mutation in *pncA* or its promoter. Furthermore, the hot spot regions identified could indicate regional convergent evolution associated with PZA resistance.

### Role of rpsA

The gene *rpsA* has held a hotly debated position within the literature, as there have been multiple publications both supporting and dismissing the gene’s role in PZA resistance^24, 26, 40, 41^ While the trans-translational function of *rpsA* was shown to be inhibited by PZA (thought to explain PZA resistance in isolates without a *pncA* mutation)^24^, Alexander *et al*. were not able to find any phenotypically informative mutations in the gene^26^. Regardless, due to low prevalence, the predictive value of *rpsA* as an indicator for PZA resistance tends to be relatively low. In this study, only three heterogeneous frameshifts in *rpsA* (-C1065, -C1142, and -CA660) could potentially hold the molecular basis for otherwise unexplained resistant cases (Supplementary Table ST2). All three were novel and their causal role in resistance needs to be confirmed. Assuming a causal role for all three mutations, the diagnostic sensitivity of RpsA was around 1% (3/224) in this study.

### Role of panD

Similarly, mutations in *panD* have been associated with PZA resistance in isolates with a WT *pncA* ^25, 30^. We only observed one such potential case. Dillon *et al*. postulated that media supplemented with pantothenate, certain pantothenate analogs, or other metabolites likely explained the PZA resistance of strains with mutations in *panD* ^27^. The authors proposed that PZA resistance was independent of mutations in *panD*.

### Causation

Generally, all changes in PZase are associated with PZA resistance^4, 12^, yet this is not true for all mutations^14, 23^. Some changes may still render a functional enzyme^42, 43^, leading to low level resistance. Understanding the effects of these changes on the enzyme is crucial. Furthermore, the prevalence of unexplained resistance cases is currently likely underestimated since laboratory confirmation of the role of most variants in resistance is still lacking. This work could identify polymorphisms that do not play a role in resistance, or cause low levels of resistance well below the cutoff, adding to the number of unexplained resistance cases. As such, the causal role of all *pncA*, *rpsA*, and *panD* mutations in resistance should be experimentally confirmed in *M. tuberculosis*, similar to studies performed on *inhA* ^44^, *katG* ^45^, *rpoB* ^46, 47^, and *gyrA* ^48, 49^.

### Alternative mechanisms

In this study, 18 PZA^R^ isolates lacked any polymorphisms in the three genes. Other studies have also reported such resistant cases^14, 23^. A complementary or alternative mechanism of resistance, other than *rpsA* and *panD*, is most frequently suspected. A complementary mechanism regulating expression of PZase would explain the resistant cases with a WT gene. *rpsA* and *panD* do not adequately address this problem since both are considered targets of POA (activated form of PZA by PZase)^24, 25, 27^. Such a mechanism has proven to be elusive in spite of efforts in a number of laboratories around the world.

### Heterogeneity

Chemotherapy in a host with mixed bacterial population selects for the resistant subpopulation^50^. Undetected heterogeneity could be an explanation for unexplained resistant cases. Using our WGS approach, we were able to detect low levels of heteroresistance, closer to the sensitivity of MGIT DST (10%)^51^. It is still possible that some unexplained resistance cases are due to existence of small resistance subpopulation detectable by DST but not by our WGS. Heterogeneity, as detected by our approach, seemed to be a random event in *panD* and *rpsA* with equal frequencies among resistant and sensitive isolates (Supplementary Table ST8). In *pncA*, however, heterogeneity had a notably higher frequency (nearly two-fold) among resistant as compared to susceptible isolates with a diagnostic specificity of 92% (Supplemental Table ST7). The association of heterogeneity with phenotypic resistance and its utility in diagnostics needs to be investigated in a larger cohort. In this study, we considered resistant cases with heterogeneous *pncA* mutations as explained cases.

## Conclusion

A diagnostic approach, based on all *pncA* mutations, seems to be more appropriate than any selective criterion suggested in the literature as a diagnostic platform would err more on the false resistance side. While *pncA* as a whole demonstrated high association with PZA phenotype, *rpsA* and *panD* did not among our isolates and elsewhere^52^. The existence of 18 PZA^R^ isolates lacking PZase activity with WT promoter and coding regions of the three genes may suggest a missing regulatory component in the currently understood mechanism of resistance. The high number of novel variations in PZase of PZA^S^ isolates may suggest an undersampling of PZA-susceptible XDR-TB isolates in sequencing. For a comprehensive picture of the *pncA* genotype, this needs to be corrected. We also demonstrated that heterogeneity in *pncA* may not be a random event and that there are lineage-specific patterns among *pncA* mutations.

Overall, the results of this study demonstrate that a molecular diagnostic platform may suffer from a notable false resistance or false susceptibility error rate among MDR- and XDR-TB cases. In high TB burden countries this would introduce a non-negligible number of misdiagnosed cases.

## Materials and Methods

### Isolate Selection

*M. tuberculosis* strains were isolated from patient sputum in four countries (India, Moldova, Philippines, and South Africa). This effort was performed as part of a separate project called the Global Consortium for Drug-resistant tuberculosis Diagnosis (GCDD)^53^. Details of patient selection and sample collection methodology are described by Garfein *et al*.^28^ and in the Supplementary Methods. All sequencing and phenotypic data was downloaded from the publically available repository on NCBI (BioProject: PRJNA353873).

### MIRU-VNTR, Spoligotyping, and Lineage Determination

Genotyping using mycobacterial interspersed repetitive units variable number of tandem repeats (MIRU-VNTR) and spoligotyping were described by Garfein *et al*.^28^. Lineage determination based on MIRU and spoligo information was also described by Garfein *et al*.^28^. A brief summary is provided in the Supplementary Methods.

### Phenotyping

All isolates were tested for phenotypic resistance to seven first- and second-line drugs, INH, RIF, three injectable antibiotics (kanamycin, amikacin, capreomycin), and two from the quinolone group of drugs (moxifloxacin and ofloxacin). DST results for these drugs have been previously published by Garfein *et al*.^28^. Standard BACTEC MGIT 960 methods were performed using WHO recommended critical concentrations.

PZA susceptibility testing was performed on BACTEC MGIT 960 for this study. Isolates with discrepant phenotypic and *pncA* genotypic results were further examined for PZase activity^54^. A brief description of both susceptibility testing and enzyme activity is located in the Supplemental Methods.

To test the validity of our phenotypic results we explored two common validation approaches: parallel and orthogonal testing. Parallel testing would require the DST to be repeated while orthogonal testing would require an independent method with an independent error profile as compared to that of MGIT DST, such as Wayne’s enzymatic assay. Its error profile is the opposite of DST: higher false sensitivity rates but much lower false resistance rates^55^. Because this study aims to identify molecular markers that can be used for diagnosis of resistance, we chose the orthogonal approach for its lower likelihood of false resistance error. A wide range of rates for false resistance has been reported for PZA DST with some as high as 60%^32, 56, 57^. Recently this rate was estimated to be at 11.3% by Murray *et al*.^58^. The parallel approach with a repeat DST, therefore, would have ~1% false resistance rate (assuming a white noise, nonsystematic, random error event—otherwise higher) when both results agree. Wayne’s assay has a 3% false resistance rate as most of its errors belong to the false sensitivity category^55^. As such, orthogonal testing has a false resistance rate of 0.3% (1/3 that of the parallel approach) when the results of both tests agree. Additionally, since it has not yet been established whether the false resistance rate of DST is a problem with the method or a characteristic of certain isolates (e.g. “flip-flopping” between multiple DST results), orthogonal testing allows the separation and investigation of the two potential causes.

### Whole-Genome Sequencing

Sample and library preparation and post-sequencing analysis are described in the Supplementary Methods. Base calling was performed by consideration of reads supporting major and minor variants. Positions with a minor variant were labeled as “heterogeneous”, otherwise, the position was considered “monoclonal”. Minor variants were called using the criterion suggested by Black *et al*.^59^. For genotypic-phenotypic analysis we considered heterogeneous populations (in positions of consequence) as resistant since a mutant subpopulation was detected.

For this study, we considered the promoter (200 base pairs transcriptionally upstream from the annotated start site) and the coding regions of three genes: *pncA* (*rv2043c*), *rpsA* (*rv1630*), and *panD* (*rv3601c*). The genome positions for the six regions based on H37Rv reference (GenBank accession NC_000962.3) are listed in Supplementary Table ST9. These regions were examined for presence of genomic variation using the variant analysis methods described above and in the Supplementary Methods.

## Electronic supplementary material

Supplementary Material

Supplementary Table ST2
