# Supplementary material for: A Multinational Analysis of Mutations and Heterogeneity in PZase, RpsA, and PanD Associated with Pyrazinamide Resistance in M/XDR Mycobacterium tuberculosis
Source: Sci Rep. 2017 Jun 19;7:3790. doi: 10.1038/s41598-017-03452-y (PMC5476565; doi:10.1038/s41598-017-03452-y)
Supplement: Supplementary file 1 — Supplementary Material [file 41598_2017_3452_MOESM1_ESM.pdf]

A Multinational Analysis of Mutations and Heterogeneity in PZase, RpsA, and PanD Associated with Pyrazinamide Resistance in M/XDR *Mycobacterium tuberculosis*

SM Ramirez-Busby, TC Rodwell, L Fink, D. Catanzaro, RL Jackson, M Pettigrove, A Catanzaro, and F Valafar

SUPPLEMENTARY MATERIAL

SUPPLEMENTARY METHODS

Isolate Selection

Isolates were drawn from TB repositories at 1) Hinduja National Hospital (PDHNH) in Mumbai, India; 2) Phthisiopneumology Institute (PPI) in Chisinau, the central unit of the National TB Control in Moldova; 3) Tropical Disease Foundation (TDF) in Manila, Philippines; and 4) The National Health Laboratory Service of South Africa (NHLS) in Johannesburg. Each isolate was extracted from the sputum of a unique pulmonary TB patient. Priority was given to MDR and XDR cases. Overall, 315 patients were chosen for this study. Details of sample collection methodology are available in Garfein et. Al.[1]

MIRU-VNTR, Spoligotyping, and Lineage Determination

Genotyping using mycobacterial intersperses repetitive units variable number of tandem repeats (MIRU-VNTR) and spoligotyping were described elsewhere.[1] Briefly, we analyzed 12 mycobacterial interspersed repetitive units (MIRU-12) assays and spoligotyping using standardized methods.[2–4] Lineage was determined by combining and uploading the spoligotype patterns and MIRU-VNTR to the web-based TB-lineage tool.[5,6] The lineage types were predicted using a knowledge-based Bayesian network for the major phylogenetic lineages.[7]

Phenotyping

PZA Drug Susceptibility Testing: DST for PZA resistance was performed on BACTEC MGIT 960 for this study and is reported here for the first time. Archived, colony-picked clinical isolates were revived by placing one *M. tuberculosis* saturated microbank bead in a MGIT 7H9 tube with 0.8ml PANTA supplement and grown until MGIT 960 flagged the culture positive. This culture was considered the seed tube for the PZA drug susceptibility assay. If the seed tube was 1-2 days post-instrument positive it was treated as a 1:5 dilution of a 0.5 McFarland standard. If the seed tube was 3-5 days post-instrument positive it was adjusted to 0.5 MacFarland using a DEN-1 densitometer (Grant Instruments, UK) diluted to 1:5 and used as the inoculum for the PZA drug susceptibility assay. The assay was performed using the BACTEC MGIT PZA Drug kit (critical concentration of 100 mg/L) and PZA medium (pH 5.9) (Becton Dickinson Diagnostic Systems, Sparks, MD) using MGIT 960 instrument with EpiCenter software, following the manufacturer's protocol.[8] *M. tuberculosis* strain H37Rv (TMC102, ATCC# 27294) was used as a PZA susceptible control. *M. tuberculosis* strain H37Rv-PZA-R (TMC 311, ATCC# 35828) was used as a PZA resistant control.

Enzymatic Assay: Isolates with discrepant phenotypic and *pncA* genotypic results were further examined for PZase activity. The PZase biochemical assay was conducted as described by Wayne[9] with the following modifications from Singh et al.[10] A higher concentration of PZA was used (400 mg/L versus 100 mg/L) and agarose was used instead of agar to improve visualization of the color change. The surface of the PZase butts were inoculated in duplicate with a visible clump of culture from LJ slants less than 3 weeks old. The PZase butts were read at 4 and 7 days by adding 1ml 1% ferrous ammonium sulfate. A pink band of any intensity was considered positive for PZase. *M. tuberculosis* strain H37Rv (TMC102, ATCC# 27294) was used as a PZase

positive control. *M. tuberculosis* strain H37Rv-PZA-R (TMC 311, ATCC# 35828) was used as a PZase negative control. Additional controls included a selection of isolates within the sample set for which the genotypic and phenotypic results agreed.

Whole Genome Sequencing

Sample preparation and DNA extraction: For WGS, 347 isolates were streaked for isolation by standard microbiological methods. Well separated colonies were selected, emulsified and subcultured to Lowenstein-Jensen slants and Middlebrook 7H11 plates. Cultures were incubated until growth of a full bacterial lawn. A portion of growth was used to prepare frozen stocks on Microbank™ Bacterial and Fungal Preservation System beads (Pro-Lab Diagnostics, Round Rock TX) for later phenotypic DST and the remainder of the culture was used for extraction of DNA. DNA was extracted using Genomic-tips (Qiagen Inc., Germantown, MD) following the manufacture's sample preparation and lysis protocol for bacteria with the following modifications. Culture was harvested directly into buffer B1/RNAse solution, homogenized by vigorous vortex mixing and inactivated at $80^{o}C$ for 1 hour. Lysozyme was added and incubated at $37^{o}C$ for 30 minutes followed by the addition of proteinase K and further incubation at $37^{o}C$ for an additional 60 minutes. Buffer B2 was added and the mixture was incubated overnight at $50^{o}C$. The remainder of the Genomic-tip protocol was carried out exactly as described by the manufacturer.[29] DNA purity and concentration was analyzed on a Nanodrop 1000 (Thermo Scientific, Waltham, MA, USA).

Library Preparation: DNA libraries for PacBio (Pacific Biosciences, Melon Park, CA) were prepared using PacBio's DNA Template Prep Kit with no follow-up PCR amplification. Briefly, sheared DNA was end repaired, and hairpin adapters were ligated using T4 DNA ligase. Incompletely formed SMRTbell templates were degraded with a combination of Exonuclease III

and Exonuclease VII. The resulting DNA templates were purified using SPRI magnetic beads (AMPure, Agencourt Bioscience, Beverly, MA) and annealed to a two-fold molar excess of a sequencing primer that specifically bound to the single-stranded loop region of the hairpin adapters. SMRTbell templates were subjected to standard SMRT sequencing using an engineered phi29 DNA polymerase on the PacBio RS system according to manufacturer's protocol.

Post sequencing Analysis: Raw H5 files produced by the RS were processed by an in-house WGS pipeline developed for the Pacific Biosciences RS system. It employs a combination of SMRT portal, Genome Analysis Toolkit,[11,12] SamTools,[13] Varscan,[14] and in-house variant calling and housekeeping. In brief, the raw H5 data files of RS were imported into SMRT Portal. Reads from the H5 files were aligned to *M. tuberculosis* H37Rv genome from NCBI by BLASR, SMRT portal’s Basic Local Alignment. Quality scores were recalibrated and converted from PacBio’s scoring metric into Phred quality scores by using GATK. SAMtools converted recalibrated BAM files into pileup files for VarScan’s consensus generator. Variants were called utilizing an in-house script.

References

1. Garfein, R. S. *et al.* Phenotypic and genotypic diversity in a multinational sample of drug-resistant Mycobacterium tuberculosis isolates. *Int J Tuberc Lung Dis* **19,** 420–7 (2015).
2. Kamerbeek, J. *et al.* Simultaneous detection and strain differentiation of Mycobacterium tuberculosis for diagnosis and epidemiology. *J. Clin. Microbiol.* **35,** 907–14 (1997).
3. Cowan, L. S. *et al.* Evaluation of a two-step approach for large-scale, prospective genotyping of Mycobacterium tuberculosis isolates in the United States. *J. Clin. Microbiol.* **43,** 688–95 (2005).
4. Mazars, E. *et al.* High-resolution minisatellite-based typing as a portable approach to global analysis of Mycobacterium tuberculosis molecular epidemiology. *Proc. Natl. Acad. Sci. U. S. A.* **98,** 1901–6 (2001).
5. Shabbeer, A. *et al.* TB-Lineage: an online tool for classification and analysis of strains of Mycobacterium tuberculosis complex. *Infect. Genet. Evol.* **12,** 789–97 (2012).
6. Aminian, M., Shabbeer, A. & Bennett, K. P. A conformal Bayesian network for classification of Mycobacterium tuberculosis complex lineages. *BMC Bioinformatics* **11 Suppl 3,** S4 (2010).
7. Aminian, M. *et al.* Predicting Mycobacterium tuberculosis complex clades using knowledge-based Bayesian networks. *Biomed Res. Int.* **2014,** 398484 (2014).
8. Srisuwanvilai, L.-O. *et al.* Performance of the BACTEC MGIT 960 compared with solid media for detection of Mycobacterium in Bangkok, Thailand. *Diagn. Microbiol. Infect. Dis.* **61,** 402–7 (2008).
9. Wayne, L. G. Simple pyrazinamidase and urease tests for routine identification of mycobacteria. *Am Rev Respir Dis.* **109,** 147–51 (1974).

10. Singh, P. *et al.* Comparative evaluation of Löwenstein-Jensen proportion method, BacT/ALERT 3D system, and enzymatic pyrazinamidase assay for pyrazinamide susceptibility testing of Mycobacterium tuberculosis. *J. Clin. Microbiol.* **45,** 76–80 (2007).

11. McKenna, A. *et al.* The Genome Analysis Toolkit: a MapReduce framework for analyzing next-generation DNA sequencing data. *Genome Res.* **20,** 1297–303 (2010).

12. DePristo, M. A. *et al.* A framework for variation discovery and genotyping using next-generation DNA sequencing data. *Nat. Genet.* **43,** 491–8 (2011).

13. Li, H. *et al.* The Sequence Alignment/Map format and SAMtools. *Bioinformatics* **25,** 2078–9 (2009).

14. Koboldt, D. C. *et al.* VarScan 2: somatic mutation and copy number alteration discovery in cancer by exome sequencing. *Genome Res.* **22,** 568–76 (2012).

SUPPLEMENTARY TABLES AND FIGURES

Supplementary Table ST1. Stratification of isolates with discordant *pncA* genotypic and PZA phenotypic based on the seven-drug phenotypic profile. Total PZA^R: 224; Total PZA^S: 72

Description	Pan-Susceptible	MDR-TB	Pre-XDR-TB	XDR-TB	Other
**PZA^R with WT *pncA* and promoter (22)**	0	1	2	19	0
**PZA^S with mutant *pncA* or promoter (25)**	3	3	3	15	1[£]

Pan-Susceptible: susceptible to seven drugs (RIF, INH, CAP, KAN, AMK, OFX, and MOX); **MDR-TB:** resistant to INH and RIF; **Pre-XDR-TB:** MDR-TB isolates that are also resistant to either a fluoroquinolone or an injectable anti-tubercular drug; **XDR-TB:** MDR-TB isolates that are also resistant to at least one fluoroquinolone and an injectable anti-tubercular drug; **Other:** Unusual phenotypic patterns such as resistant to RIF and MOX but susceptible to all other study drugs; **WT:** Wild type.

[£] Susceptible to RIF and PZA but resistant to all others

Supplementary Table ST2. Comprehensive list of all isolates including their mutations in *pncA*, *rpsA*, and *panD*, phenotypic DST, PZase activity, and their lineage. Please see the file Supplementary Table S2.xlsx.

Supplementary Table ST3. Frequency of novel *pncA* (coding and promoter) polymorphisms in PZA resistant clinical *M. tuberculosis* isolates

Mutation	Number of Resistant	Mutation	Number of Resistant	Mutation	Number of Resistant
-A169	1	-CC509	1	Gly105Ser	1
-A187	2	-CCACA184	1	Gly97Val	1
-A28	2	-CG525	1	Phe58Val	1
-A402	1	-CGGC338	1	Ser59Phe	1
-A421	1	-CTT281	1	Ser66STOP	3
-A459	1	Cys14STOP	1	-T172	1
-A528	2	-G236	1	-T277	1
-AC528	1	-G275	1	-T463	1
-ACC344	1	-G289	2	-T475	1
Asp145Asn	1	-G313	1	-T523	2
Asp158Val	1	-G370	1	-T544	1
-C-126	1	-G526	1	-T549	1
-C-128	1	-GGCC178	1	Thr47Ile	1
-C420	1	-GGTCGCGCCGCT516	1	+TT-99	1
-CACC345	2				

Supplementary Table ST4. Frequency of novel *pncA* (coding and promoter) polymorphisms in PZA susceptible clinical *M. tuberculosis* isolates

Mutation	Number of Susceptible
**Tyr41Asp**	1
**-T-161**	1
**-G519**	1
**Asp136Ala**	1
**+CG508**	1
**+C507**	1
**Ala165Gly**	1
**-T558**	1
**G-5C**	1
**Ser59Phe**	1
**-G322**	1
**Val9Ala**	1
**Phe58Val**	1
**+G559**	1
**Phe106Leu**	1

Supplementary Table ST5. PZA^R isolate counts stratified based on lineage and mutations observed in seven regions of *pncA* gene. Total number of resistant isolates in each lineage is given in parentheses in each column header. "< 0" indicates the group of isolates from Supplementary Figure 1A in the promoter region of the gene. For example, two isolates ($\frac{2}{81} \approx 2$%) from Euro-American lineage harbored a promoter mutation. "No Mutation" is the group of isolates that had a WT PZase and *pncA* promoter. Red font indicates an unusually high percentage. Green font indicates unusually low percentage.

Codon	Euro-American (81)	East Asian (Beijing) (115)	Indo-Oceanic (18)	East-African Indian (CAS) (9)	East-Asian (1)
**< 0**	2 (2%)	5 (4%)	0 (0%)	0 (0%)	0 (0.00%)
**1-30**	10 (12%)	37 (32%)	1 (6%)	3 (33%)	0 (0.00%)
**31-60**	10 (12%)	3 (3%)	3 (17%)	1 (11%)	0 (0.00%)
**61-90**	11 (14%)	12 (10%)	4 (22%)	0 (0%)	0 (0.00%)
**91-120**	4 (5%)	14 (12%)	1 (6%)	1 (11%)	0 (0.00%)
**121-150**	12 (15%)	19 (17%)	6 (33%)	2 (22%)	1 (100%)
**151-187**	20 (25%)	17 (15%)	2 (11%)	1 (11%)	0 (0.00%)
**Total**	69 (85%)	107 (93%)	17 (94%)	8 (89%)	1 (100%)
**No Mutation**	12 (15%)	8 (7%)	1 (6%)	1 (11%)	0 (0%)

Supplementary Table ST6. Lineage-based stratification of *M. tuberculosis* isolates that harbored the most frequently observed *rpsA* mutation (the synonymous Arg212Arg)

Lineage	Number of PZA^R	Number of PZA^S
East-Asian (Beijing)	95 (83%)	20 (95%)
East-Asian	1 (100%)	0
Euro-American	3 (4%)	0
Indo-Oceanic	0	0
East-African Indian (CAS)	0	0

Supplementary Table ST7. Number of isolates with a heterogeneous polymorphism in 224 PZA^R and 72 PZA^S isolates, by gene

Gene	PZA^R (224)	PZA^S (72)
pncA	34(15%)	6 (8%)
rpsA	16 (7%)	5 (7%)
panD	8 (4%)	3 (4%)

Supplementary Table ST8. Heterogeneous mutations in *pncA* of PZA^R *M. tuberculosis* clinical isolates that have no other nonsynonymous coding or promoter mutations in *pncA*, *panD*, and *rpsA*

Mutation	Number of PZA^R	Number of PZA^S
**Gly97Val/-G289**	1	0
**+GG388**	1	0
**+G516**	1	0
**Gly132Ala**	1	0
**+C453***	5	0
**+GG390**	1	0
**-G313***	1	0
**Gly97Ser**	1	0
**Leu182Ser**	1	0

Supplementary Table ST9. The start and end positions of the regions of *M. tuberculosis* H37Rv genome (GenBank accession NC_000962.3) used for the analysis of the three genes *pncA*, *rpsA*, and *panD*.

	***pncA* promoter**	***pncA* (Rv2043c)**	***rpsA* promoter**	***rpsA* (Rv1630)**	***panD* promoter**	***panD* (Rv3601c)**
**Starting position**	2289241	2288681	1833342	1833542	4044281	4043862
**End position**	2289441	2289241	1833542	1834987	4044481	4044281
**Length (bp)**	200	561	200	1446	200	420

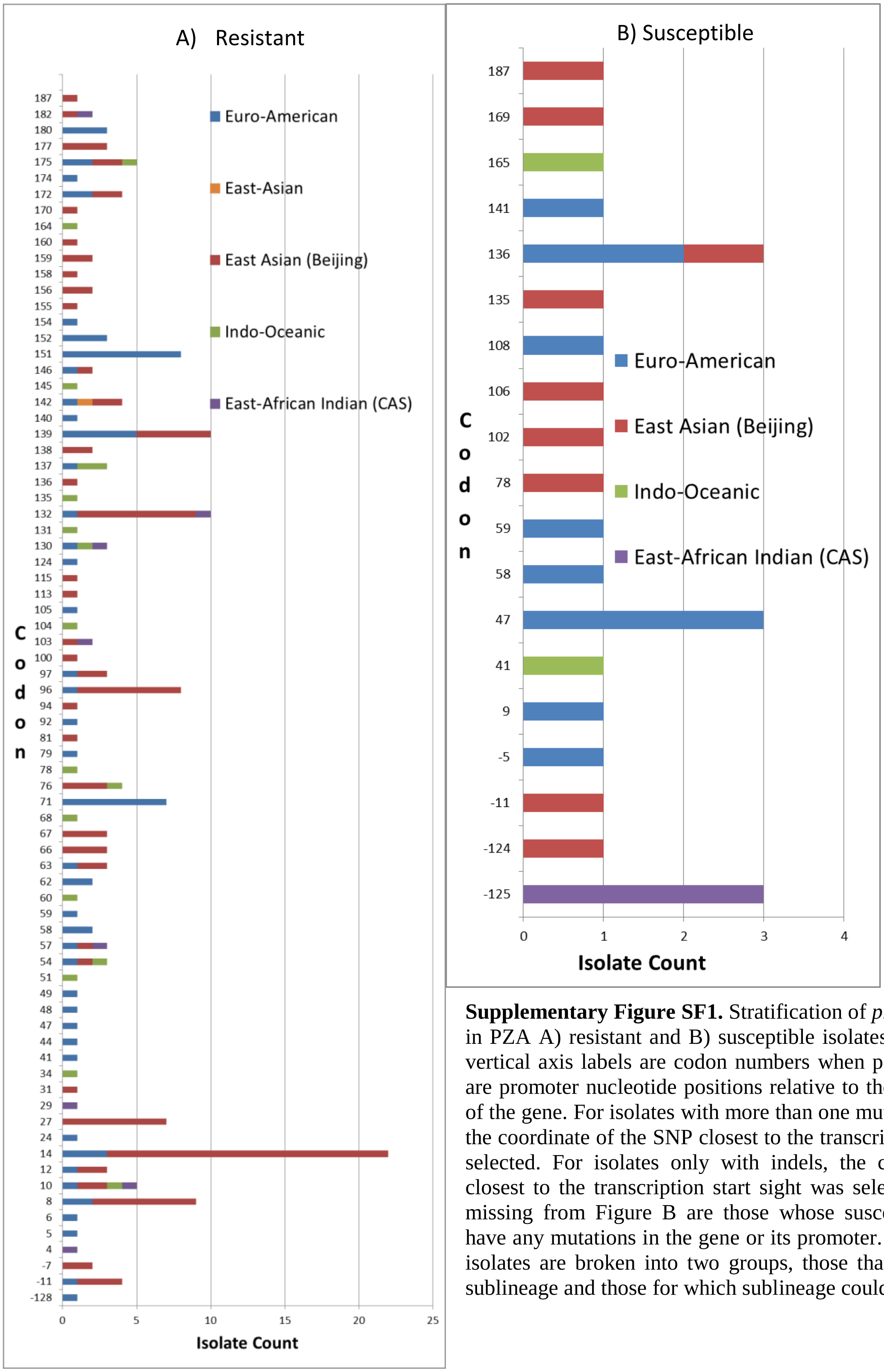

Supplementary Figure SF1. Stratification of *pncA* mutations observed in PZA A) resistant and B) susceptible isolates based on lineage. The vertical axis labels are codon numbers when positive. Negative labels are promoter nucleotide positions relative to the transcription start site of the gene. For isolates with more than one mutation, if a SNP present, the coordinate of the SNP closest to the transcription start site has been selected. For isolates only with indels, the coordinate of the indel closest to the transcription start sight was selected. Lineages that are missing from Figure B are those whose susceptible isolates did not have any mutations in the gene or its promoter. Lineage 2 (East-Asian) isolates are broken into two groups, those that belong to the Beijing sublineage and those for which sublineage could not be determined.

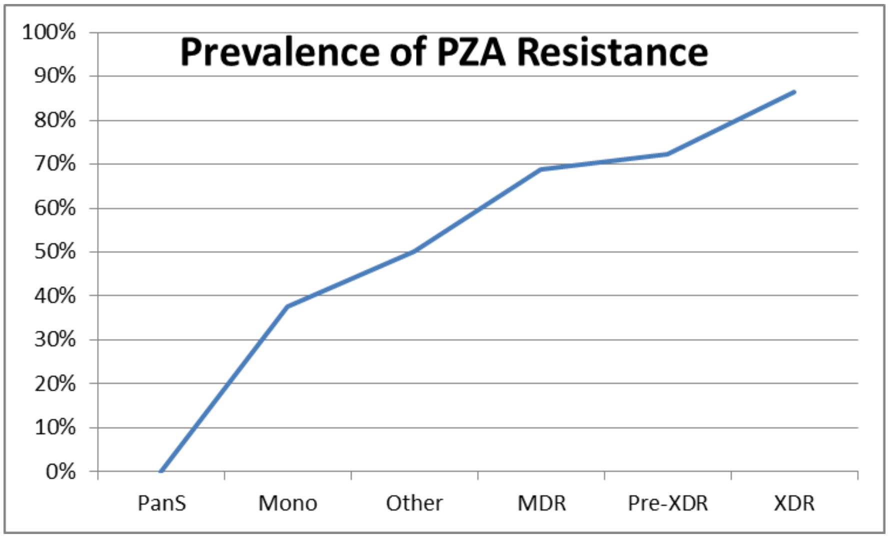

Supplementary Figure SF2. Prevalence of PZA resistance with respect to resistance to seven other study drugs (RIF, INH, CAP, KAN, AMK, OFX, and MOX). **PanS:** susceptible to all seven drugs; **MDR-TB:** resistant to INH and RIF; **Pre-XDR-TB:** MDR-TB isolates that are also resistant to either a fluoroquinolone or an injectable anti-tubercular drug; **XDR-TB:** MDR-TB isolates that are also resistant to at least one fluoroquinolone and an injectable anti-tubercular drug; **Other:** Unusual phenotypic patterns such as resistant to RIF and MOX but susceptible to all other study drugs.
